# Responsibility, opportunity, and vision for higher education in urban and regional carbon management

**DOI:** 10.1186/1750-0680-1-13

**Published:** 2006-11-21

**Authors:** Penelope Canan, Erich W Schienke

**Affiliations:** 1 Department of Sociology, University of Central Florida, Orlando, Florida, USA; 2 Rock Ethics Institute, Pennsylvania State University, University Park, Pennsylvania, USA

## Abstract

This is a summary of the conversation among scholars attending the special session on "Responsibility, Opportunity, and Vision for Higher Education in Urban and Regional Carbon Management" at the First International Conference on Carbon Management at Urban and Regional Levels: Connecting Development Decisions to Global Issues in Mexico City Sept. 4–8, 2006. It includes The Declaration for Carbon Management Education, agreed upon by the participants. Obstacles to such a vision were discussed along with exemplar models of transdisciplinary curricula and suggestions for scholarship.

## Introduction

The third theme of the Science Framework of the Global Carbon Project is Carbon Management [1]. At the GCP's first conference dedicated to the topic, Carbon Management at Urban and Regional Levels: Connecting Development Decisions to Global Issues in Mexico City Sept. 4–8, 2006, educators and students met at a Special Afternoon Session for Higher Education. This session was framed as a discussion on "Responsibility, Opportunity, and Vision for Higher Education in Urban and Regional Carbon Management." Its goals were to "explore *Earth System Science *course content and our own programmatic challenges/weaknesses/strengths and then to move on to envisioning innovations such as inter-collegiate team taught virtual seminars, visitor training exchanges for graduate students, and opportunities for collaborative hands-on projects in carbon science and management" [2].

## Discussion

In 2001, the scientific communities of four international global change research programs – the International Geosphere-Biosphere Programme (IGBP), the International Human Dimensions Programme on Global Environmental Change (IHDP), the World Climate Research Programme (WCRP) and the international biodiversity program DIVERSITAS – concluded that, in addition to the threat of significant climate change,

...there is growing concern over the ever-increasing human modification of other aspects of the global environment and the consequent implications for human well-being. Basic goods and services supplied by the planetary life support system, such as food, water, clean air and an environment conducive to human health, are being affected increasingly by global change [3].

### The challenge of the Amsterdam declaration of 2001

Accordingly, the four programs called for an "ethical framework for global stewardship and strategies for Earth System management... that sustain the Earth's environment while meeting social and economic development objectives" [3]. Furthermore, they urged embarking on a new system of global environmental science, an Earth Systems Science, which would

• draw strongly on the existing and expanding disciplinary base of global change science;

• integrate across disciplines, environment and development issues and the natural and social sciences;

• collaborate across national boundaries on the basis of shared and secure infrastructure;

• intensify efforts to enable the full involvement of developing country scientists; and

• employ the complementary strengths of nations and regions to build an efficient international system of global environmental science.

The session at the Mexico City conference was initiated in response to the challenge of the Amsterdam Declaration.

### Report from the special afternoon session for higher education in urban and regional carbon management

The president of Oklahoma State University USA, Dr. David Schmidly, began the afternoon by outlining the challenge of education in sustainability for research universities [4]. The following discussion revealed that the participants represented a wide variety of disciplinary perspectives and levels of institutional support for education relevant to carbon management. Furthermore, the participants stressed the importance of practical teamwork as a model for the kind of transdisciplinary curricular innovation that is required for carbon management education (Table 1).

**Table 1:** Summary of Discussion Topics and Suggestions for Curricular Innovations for Carbon Management in Higher Education

▪ A variety of specialization – from meteorology, to oceanography, geography, forestry, sociology, and ethics;
▪ Differences in support for and ease of transdisciplinary boundary crossing and innovation;
▪ Wide variation in financial resources for education and research and a universal need for cross-cultural opportunities for graduate education;
▪ Many varieties of cross-cultural exchange that are particular to specific bilateral university arrangements;
▪ The need for intellectual leadership to guide innovation in higher education;
▪ The need for ethical training, citizen responsibility, and environmental awareness within every discipline;
▪ The benefit of goal-oriented research considering the urgency of the questions at hand and the wealth of information already obtained;
▪ The need for meta-analysis of case studies concerning carbon drivers and drivers of de-carbonization;
▪ The desirability of research teams engaged in comparative case studies;
▪ The benefit of a template (or protocol) for comparative case studies; and,
▪ The opportunity for American universities to participate in the education-based climate change initiative *FocusTheNation.org *culminating in January 2008.

Based on this conversation, the participants committed themselves to mutual support and communication, enlarging the nascent network of educators, connecting with other similar networks, and pushing for institutional changes to further such goals. The resulting position statement was adopted as the Mexico City Declaration on Carbon Management Education (Table 2). The signatories are listed in Table 3.

**Table 2:** Mexico City Declaration on Carbon Management Education

• *Whereas*, the carbon-climate-human cycle presents a major challenge for all sectors of society and all places in the world, now and in the future;
• *Whereas*, the complexity of the carbon-climate-human cycle is extremely high and requires the combination and integration of many relevant scientific disciplines and management perspectives in higher education;
• *Whereas*, adaptation and mitigation of climate change impacts will require a new type of expertise and citizens knowledgeable about the carbon-climate-human cycle;
• *Whereas*, the Amsterdam Declaration of 2001 called for *an ethical framework for global stewardship and strategies for Earth System management*, and *a new system of global environmental science known as Earth Systems Science*;
• *Whereas*, our institutions of higher education are responsible for providing tomorrow's leaders with the training in "earth system science," and
• *Whereas*, most institutions of higher education have been organized in a manner that does not foster research and training on the carbon-climate-human cycle nor facilitates the types of exchanges among faculty or across faculty and students from around the world;
• *Therefore*, we call for programmatic innovations in institutions of higher education in their reward structures such that they facilitate and promote transdisciplinary carbon management research and education for global environmental citizenship and ethical leadership that includes:
• Increased opportunities for transdisciplinary symposia and seminars on the carbon-climate-human cycle;
• Alteration of tenure criteria that is narrowly discipline-based to one that is truly open to and encouraging of interdisciplinarity;
• Change in university accounting methods away from those that encourage turf protection which in turns discourages interdisciplinarity;
• The encouragement of integrative goal-based case study team-work for students where transdisciplinarity becomes evident in the field with immediate and transparent educational return;
• A drastic overhaul of cross-cultural educational opportunities that support the exchange of students across cultural, developmental, and geographical contexts;
• The strengthening and further development of Earth System Science and cross-cultural opportunities by all national academies of science and national funding institutions;
• Support for urban and regional carbon management projects by national academies of sciences and national research funding agencies.
• Active support for scientific publications in developing nations in SCI journals and the on-line journal of *Carbon Balance and Management *(CBM).
Drafted on September 7, 2006 at the Special Session on "Responsibility, Opportunity, and Vision for Higher Education in Urban and Regional Carbon Management" at the First International Conference on Carbon Management at Urban and Regional Levels: Connecting Development Decisions to Global Issues (Global Carbon Project), Mexico City, Mexico.

**Table 3:** Signatories to the Mexico City Declaration on Carbon Management Education

Penelope Canan, Sociology, University of Central Florida, USA, pcanan@mail.ucf.edu
Elizabeth Caniglia, Sociology, Oklahoma State University, USA, canifli@okstate.edu
Craig Coleman, Oceanography, University of Hawaii, craigc@hawaii.edu
Ben de Joug, ECOSUR, bjong@vhs.ecosur.mx
Philip Emmi, Architecture, University of Utah, USA, emmi@arch.utah.edu
Jose Martin Hernandez-Ayon, University of Baja Col ITO, jmartin@uabc.mx
Lorenz Magaard, Oceanography, International Center for Climate and Society, University of Hawaii, USA lorenz@hawaii.edu
Paul Isolo Mukwaya, Geography, Makerere University, Uganda mukwaya@arts.mak.ac.ug
Jose Navar, Forestry Sciences, IPN-Durango, Mexico, josejesusnavar@yahoo.com.mx
Rob Neff, Geography, University of Maryland Baltimore County USA, neff@umbc.edu
Marcelo Olguin, Natural Resource Management, ECOSUR, Mexico, molguin@vhs.ecosur.mx
Erich Schienke, Rock Ethics Institute, Penn State University, USA, erich@psu.edu
Chuluun Togtokh, Geography, National University of Mongolia, Mongolia, chuluun@nrel.colostate.edu
Pablo Trucco, Oceanography, University of Baja California, Mexico, mahabrana@yahoo.com.mx
Mauricio Osses, Mechanical Engineering and Modelling, Universidad de Chile, Chile, maosses@ing.uchile.cl
Brent Yarnal, Geography, Penn State University, USA, byarnal@psu.edu

**Figure 1:**
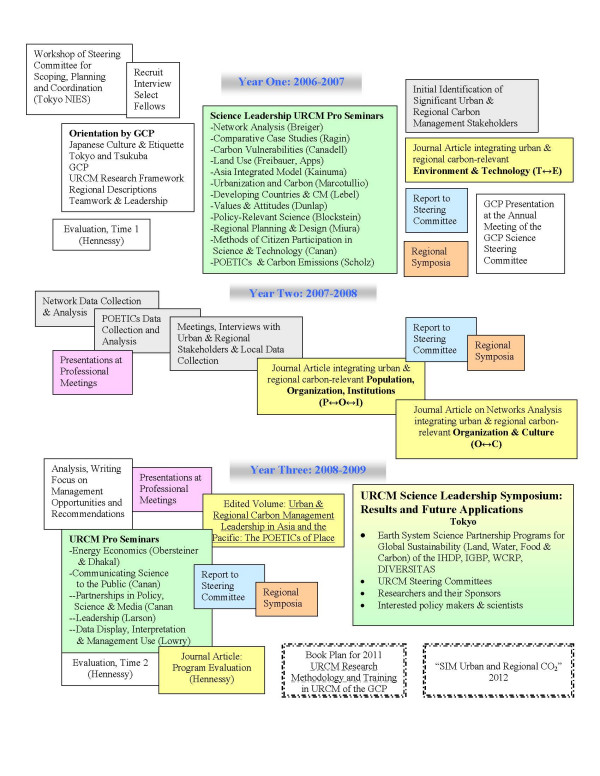
Schematic of the Proposed Science Leadership Program, Urban and Regional Carbon Management, Global Carbon Project, NIES, Tsukuba, Japan, August 2005.

An illustration of a three-year integrated, case-study based program in the social sciences is presented in Figure 1, taken from a GCP proposal for five post doctoral students from the Asia and Pacific region [5]. The organizing principle is the management challenge of finding locally appropriate levers for carbon management, given the configuration of population, (social) organization, eco-environment, technology, institutions, and culture (POETICs) in each fellow's urban or regional management case study [6].

## Conclusion

While we are only at the very beginning of contemplating graduate education in carbon management at research universities and scientific institutes, leaders in higher education understand that their commitment to the project is an important first step. The small network begun in at the Mexico City conference has pledged to create concrete next steps. Some of these await the organization of coordinated case studies, programmatic development, and curricular design. We urge our colleagues to join us as we go forward. We also hope that colleagues will use this journal to present ideas for interdisciplinary carbon balance education.

## Competing interests

Penelope Canan serves on the editorial board of the Journal of Carbon Balance and Management.
